# Transcriptomics of *Haemophilus* (*Glässerella*) *parasuis* serovar 5 subjected to culture conditions partially mimetic to natural infection for the search of new vaccine antigens

**DOI:** 10.1186/s12917-018-1647-1

**Published:** 2018-11-06

**Authors:** Álvaro Álvarez-Estrada, César B. Gutiérrez-Martín, Elías F. Rodríguez-Ferri, Sonia Martínez-Martínez

**Affiliations:** Microbiology & Immunology Section, Animal Health Department, Faculty of Veterinary Medicine, University of León, León, Spain

**Keywords:** *Haemophilus* (*Glässerella*) *parasuis*, Glässer’s disease, Vaccine antigens, RNA-sequencing, Transcriptome, Iron uptake

## Abstract

**Background:**

*Haemophilus* (*Glässerella*) *parasuis* is the etiological agent of Glässer’s disease in pigs. Control of this disorder has been traditionally based on bacterins. The search for alternative vaccines has focused mainly on the study of outer membrane proteins. This study investigates the transcriptome of *H.* (*G.*) *parasuis* serovar 5 subjected to in vitro conditions mimicking to those existing during an infection (high temperature and iron-restriction), with the aim of detecting the overexpression of genes coding proteins exposed on bacterial surface, which could represent good targets as vaccine candidates.

**Results:**

The transcriptomic approach identified 13 upregulated genes coding surface proteins: TbpA, TbpB, HxuA, HxuB, HxuC, FhuA, FimD, TolC, an autotransporter, a protein with immunoglobulin folding domains, another large protein with a tetratricopeptide repeat and two small proteins that did not contain any known domains. Of these, the first six genes coded proteins being related to iron extraction.

**Conclusion:**

Six of the proteins have already been tested as vaccine antigens in murine and/or porcine infection models and showed protection against *H.* (*G.*) *parasuis*. However, the remaining seven have not yet been tested and, consequently, they could become useful as putative antigens in the prevention of Glässer’s disease. Anyway, the expression of this seven novel vaccine candidates should be shown in other serovars different from serovar 5.

**Electronic supplementary material:**

The online version of this article (10.1186/s12917-018-1647-1) contains supplementary material, which is available to authorized users.

## Background

*Haemophilus* (*Glässerella*) *parasuis* is a Gram-negative bacterium which forms part of the microbiota in the upper respiratory tract in pigs. Under certain conditions, such as stress or absence of prior contact, virulent strains can cause a systemic infection resulting in polyserositis, meningitis or arthritis (Glasser’s disease) [1]. In addition, *H.* (*G.*) *parasuis* is involved in pneumonias as secondary agent within the porcine respiratory complex disease [2]. Each year *H.* (*G*.) *parasuis* causes significant loss to the swine industry worldwide [1].

Most vaccines used to prevent *H.* (*G.*) *parasuis* infection are bacterins although a minory of them are based on live vaccines. These traditional vaccines present several disadvantages, with the main one being the lack of cross-protection against different serotypes [3]. The use of these vaccines has been gradually replaced by subunit vaccines, whose study has been focused on outer membrane proteins (Omps) among other molecules. However, a huge variability has been still found among isolates from different countries, with substantial variations in MLST profiles, in such a manner that problems with cross-protection remain [4]. Recent advances in genomics, proteomics and transcriptomics have greatly enabled the search for Omps that are more likely to behave as good vaccine antigens [5].

Most bacteria remodel their coating structures inside the host since they need to adapt to new environments that could be potentially harmful to them, such as high temperature, osmolarity, pH or oxidative stress and these changes often involve the synthesis of surface structures that are important virulence factors [6]. It has been speculated that the change from a physiological temperature to a higher one (similar to that hyperthermia measured during Glässer’s disease) in the host could also be used by some pathogens as a signal to enter into a persistence state in animals that leads to expression of mechanisms triggered during hyperthermia, used to avoid the host immune response. Some of them may correspond to changes in bacterial surface proteins [7].

Although iron is an essential element for organisms, being required for energy processes and DNA, protein or sugar metabolism; however, the concentration of free iron in the host is not enough to support the growth of bacteria [8]. For this reason, pathogenic bacteria have developed different mechanisms to scanvenge iron from host (siderophores, hemophores or host-molecule-binding proteins), which involve the expression of surface-exposed proteins [9]. In this respect, some reports have already concerned the expression of genes of *H.* (*G.*) *parasuis* to iron-restriction stress [10–12].

The aim of this work was to study the modifications which occur in the transcriptome of *H.* (*G.*) *parasuis* by RNA sequencing, when it is grown in vitro under culture conditions of iron-restriction and temperature stresses. These conditions were selected in order to partially mimic the host environment during natural infection. The transcriptome of bacteria grown under these conditions was compared with that of bacteria grown under optimal in vitro conditions (37 °C and non-iron-restriction stress) for detecting the overexpression of genes coding proteins exposed on the bacterial surface.

## Results

### Quality control of RNA samples

The RNA integrity from each sample was tested by automated electrophoresis in a Bioanalyzer Agilent 2100. The RNA integrity number (RIN) was not calculated because of the peculiar arrangement of the rRNA peaks from bacteria belonging to genus *Haemophilus* [22], in which the 23S subunit of the rRNA is fragmented in 1.2 and 1.7 kb portions. However, the graphs showed that the RNA present in each sample had A correct integrity (data not shown).

### Upregulation under mimetic conditions (iron-restriction and 41 °C)

**Fig. 1:**
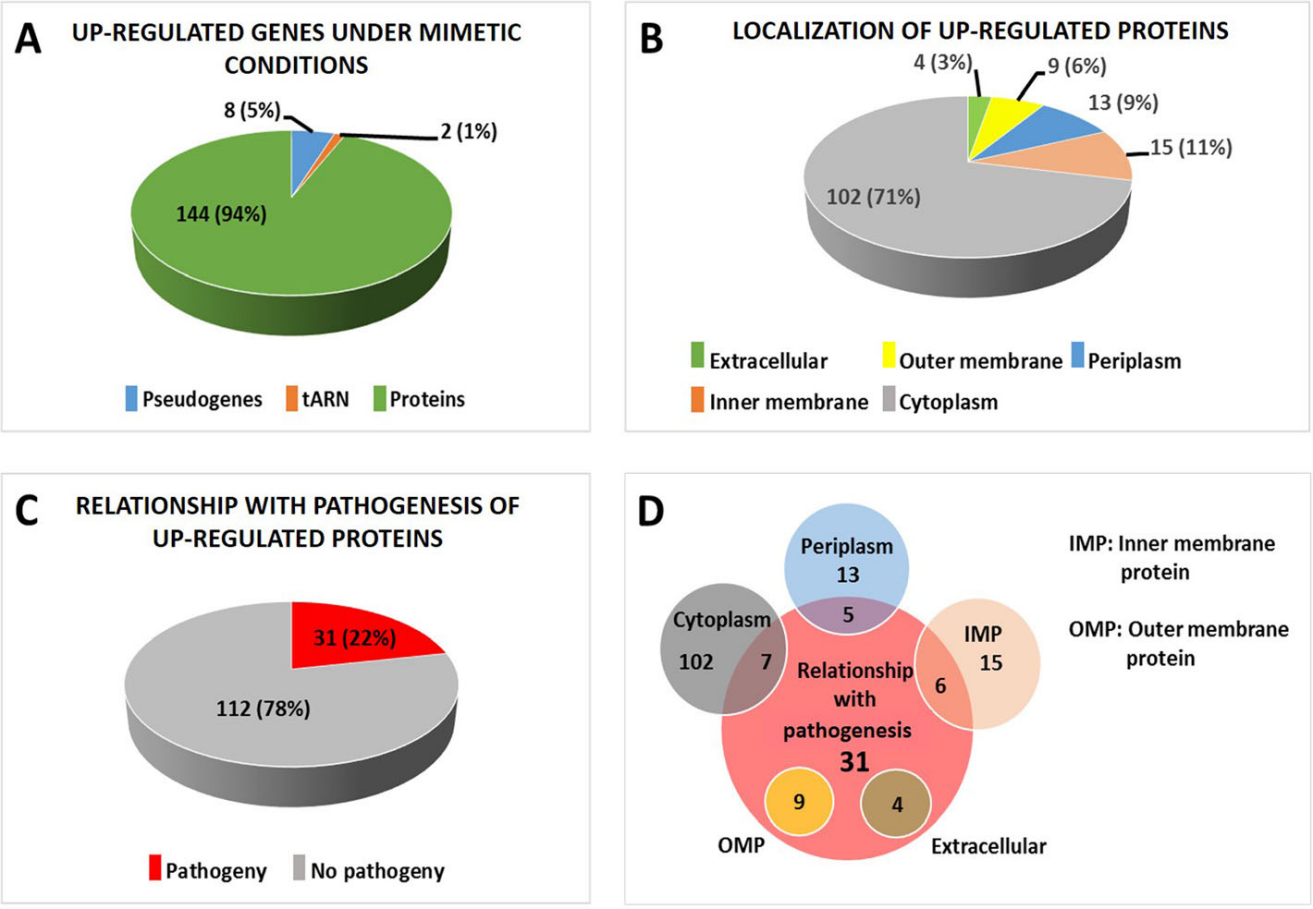
Genes and proteins upregulated under mimetic conditions. **(a)**: Number of pseudogenes, tRNA and protein-coding genes indicating the percentage of the total number of upregulated genes with log_2_ (fold change) > 10 under mimetic conditions. **(b)**: Number of different locations of proteins upregulated under mimetic conditions and percentage of total proteins. Further corrections were taken into account. **(c)**: Number and percentage of proteins related to pathogenesis upregulated under mimetic conditions. Further corrections were considered. **(d)**: Venn diagram representing the relationship between cell localization and the pathogenesis of upregulated proteins. Further corrections were taken into account

The number of genes upregulated under these conditions was 433, of which 154 had a log_2_ > 10. Among these 154, there were eight pseudogenes, two genes encoding tRNA and 144 genes encoding proteins (Fig. 1). The amino acid sequence of the proteins encoded by the upregulated genes was obtained, and the cellular location of the proteins and their relation to pathogenesis was investigated. Four extracellular proteins (Eps), 17 Omps, 10 periplasmic proteins (Pps), 13 inner membrane proteins (Imps) and 100 cytoplasmic proteins (Cps) were found using CELLO v.2.5. As the main aim of this study was to search the proteins exposed to the cell surface, we verified individually the location of those proteins that CELLO assigned as belonging to the extracellular and Omp fractions and they were found different. Thus, two of the proteins firstly assigned to Omps were found to be Imps, while three others were Pps and two more were Cps. One of the Eps and another protein initially assigned to Omps appeared to be really the same extracellular protein but they were noted as two different proteins in the reference genome because of a point mutation involving the emergence of a stop codon. Therefore, after this correction, four proteins remained assigned to an extracellular localization (Eps), nine as Omps, 15 as Imps, 13 as Pps and 102 as Cps (Fig. 1).

A total of 31 proteins were recognized as being related to pathogenesis and therefore possibly involved in the pathogenesis of Glässer’s disease (Fig. 1), and of them, four were Eps, nine were Omps, six were Imps, five were Pps and seven were Cps. All proteins predicted to be located on the bacterial surface were also predicted as being related to pathogenesis.

Table 1 shows the upregulated proteins that were predicted to be located on the bacterial surface (Omps or Eps) and/or related to pathogenesis. The proteins predicted to be located on the bacterial surface or related to pathogenes is that were not identified in GenBank and Uniprot databases were subjected to further studies of sequence homology and searched for the presence of domains of a known function. The findings are shown in Table 2. Additional file 1 summarizes the findings for genes that were upregulated under mimetic conditions with a log_2_ (fold change) > 10.

**Table 1:** Upregulated proteins under mimetic conditions related to pathogenesis and located on the bacterial surface

*Locus* ^*a*^	GenBank product	Access number Uniprot	Name Uniprot	Pathogenesis	Location
*HAPS_RS00370*	protein TolA	B8F375	|tolA|Cell envelope integrity inner membrane protein TolA	P	IM
*HAPS_RS00485*	TonB-dependent receptor	–	–	P	OM
*HAPS_RS00735*	hypothetical protein	–	–	P	OM
*HAPS_RS00740* ^*a*^	autotransporter domain-containing protein	–	–	P	OM^a^
*HAPS_RS00745* ^a^	hypothetical protein	–	–	P	EX
*HAPS_RS01255*	ABC transporter permease	B8F3P0	|HAPS_0253|ABC-type nitrate/sulfonate/bicarbonate transport permease	P	IM
*HAPS_RS01260*	ABC transporter substrate-binding protein	B8F3P1	|HAPS_0254|ABC-type nitrate/sulfonate/bicarbonate transport systems periplasmic components protein	P	CP
*HAPS_RS01265*	ABC transporter ATP-binding protein	B8F3P2	|HAPS_0255|ABC-type nitrate/sulfonate/bicarbonate transport system, ATPase	P	IM
*HAPS_RS01400*	sulfurtransferase FdhD	–	–	P	CP
*HAPS_RS01435*	hypothetical protein	B8F3S4	|HAPS_0289|Uncharacterized protein	P	CP
*HAPS_RS01805*	ABC transporter ATPase	B8F3Z5	|HAPS_0364|ATPase components of ABC transporters with duplicated ATPase domains-containing protein	P	PP
*HAPS_RS01895*	hypothetical protein	B8F410	|HAPS_0382|Uncharacterized protein	P	EX
*HAPS_RS02610*	hypothetical protein	B8F4D5	|purL|Phosphoribosylformylglycinamidine synthase	P	CP
*HAPS_RS03735*	fimbrial usher protein	–	–	P	OM
*HAPS_RS04480*	hypothetical protein	–	–	P	PP
*HAPS_RS04485*	hypothetical protein	B8F5E9	|HAPS_0923|Uncharacterized protein	P	EX
*HAPS_RS06520*	hypothetical protein	B8F6H3	|yaaH|Permease, Inner membrane protein yaaH	P	IM
*HAPS_RS00370*	protein TolA	B8F375	|tolA|Cell envelope integrity inner membrane protein TolA	P	IM
*HAPS_RS00485*	TonB-dependent receptor	–	–	P	OM
*HAPS_RS00735*	hypothetical protein	–	–	P	OM
*HAPS_RS00740* ^a^	autotransporter domain-containing protein	–	–	P	OM
*HAPS_RS00745* ^a^	hypothetical protein	–	–	P	EX
*HAPS_RS06610*	cell envelope protein TonB	B8F6J2	|tonB|Protein TonB	P	PP
*HAPS_RS07630*	iron ABC transporter permease	B8F723	|hmuU|Hemin transport system permease protein HmuU	P	IM
*HAPS_RS07950*	hypothetical protein	–	–	P	CP
*HAPS_RS09000*	hypothetical protein	B8F7Q0	|HAPS_1850|Uncharacterized protein	P	IM
*HAPS_RS10195*	DUF262 domain-containing protein	B8F899	|HAPS_2100|Uncharacterized protein	P	CP
*HAPS_RS10530*	TolC family protein tolC	B8F8F2	|tolC|RND efflux system outer membrane lipoprotein/RND superfamily resistance-nodulation-cell division antiporter	P	OM
*HAPS_RS10585*	ligand-gated channel	B8F8G2	|hxuC|Heme/hemopexin utilization protein C/outer membrane receptor protein, mostly Fe transport	P	OM
*HAPS_RS10590*	ShlB/FhaC/HecB family hemolysin secretion/activation protein	B8F8G3	|hxuB|Heme/hemopexin-binding protein B, hemolysin activation/secretion protein	P	OM
*HAPS_RS10595*	hypothetical protein	B8F8G4	|hxuA|Heme/hemopexin-binding protein A (Heme:hemopexin utilization protein A)	P	EX
*HAPS_RS10780*	membrane protein	B8F8J9	|HAPS_2219|Possible outer membrane protein/FOG: TPR repeat protein	P	OM
*HAPS_RS10800*	transferrin-binding protein-like solute binding protein	–	–	P	OM
*HAPS_RS10805*	lactoferrin/transferrin family TonB-dependent receptor	B8F8K4	|tbpA|Transferrin-binding protein 1	P	OM
*HAPS_RS11010*	transpeptidase	B8F8P5	|HAPS_2267|Uncharacterized protein	P	PP
*HAPS_RS11030*	transcriptional regulator	B8F8P9	|impA|SOS-response transcriptional repressor	P	CP

*P* indicates the relationship to pathogenesis; Locations are indicated by: *OM* outer membrane, *EX* extracellular, *IM* inner membrane, *PP* periplasmic, *CP* cytoplasmic. The *locus* in the reference genome (SH0165 strain) is indicated. ^a^ indicates that this was a single protein recognized as two different ones.

**Table 2:** Findings in upregulated proteins under mimetic conditions related to pathogenesis but not characterized in the databases

*Locus*	Access number Uniprot	Location	Findings
*HAPS_RS00735*	–	OM	Nothing was recognized
*HAPS_RS00740* ^a^	–	OM^a^	Half C-terminal Carrier ^a^
*HAPS_RS00745* ^a^	–	EX^a^	Half n-terminal auto transporter ^a^
*HAPS_RS01435*	B8F3S4	CP	Nothing was recognized
*HAPS_RS01895*	B8F410	EX	Nothing was recognized
*HAPS_RS04480*	–	PP	Domain of the superfamily Glycoside-hydrolase
*HAPS_RS04485*	B8F5E9	EX	Immunoglobulin-like fold domain
*HAPS_RS07950*	–	CP	Domain of the Thioredoxin-like superfamily
*HAPS_RS09000*	B8F7Q0	IM	Homology with permease
*HAPS_RS10195*	B8F899	CP	ParB-like and HNH nuclease domains

The locus in the reference genome (SH0165 strain) is also indicated. ^a^ indicates that this was a single protein recognized as two different ones

### Downregulation under mimetic conditions (without iron-restriction and 37 °C)

**Fig. 2:**
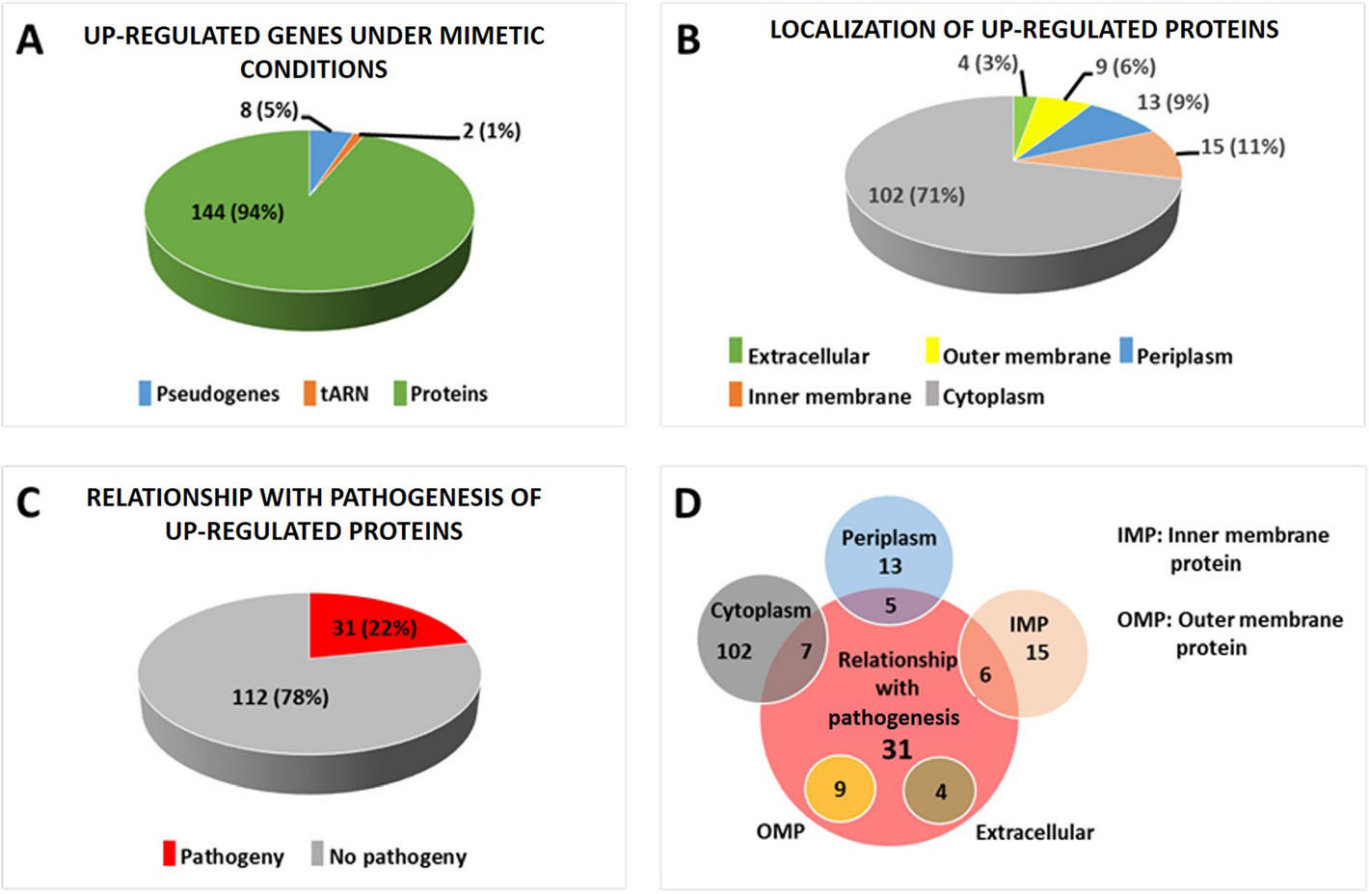
Genes and proteins downregulated under mimetic conditions. (**a)**: Number of pseudogenes, tRNA and protein-coding genes indicating the percentage of the total number of genes down regulated genes with log_2_ (fold change) > 10 under mimetic conditions. **(b)**: Number of different locations of proteins downregulated under mimetic conditions and percentage of total proteins. Further corrections were taken into account. **(c)**: Number and percentage of proteins related to pathogenesis downregulated under mimetic conditions. Further corrections were considered. **(d)**: Venn diagram representing the relationship between cell localization and the pathogenesis of downregulated proteins. Further corrections were taken into account

The number of genes underexpressed under control conditions was 460, of which 187 were selected for having a log_2_ > 10. They included four pseudogenes, seven genes encoding tRNA, another gene encoding rRNA and 175 genes encoding proteins (Fig. 2). The CELLO v.2.5 program predicted six Eps, five Omps, 34 Imps, 30 Pps and 100 Cps. Those assigned as Omps or Eps were individually rechecked and, after correction, four proteins remained assigned as Omps wihle the number of Cps rose to 101 (Fig. 2). The MP3 server found 34 proteins that were recognized as being related to pathogenesis (Fig. 2), of which five were Eps, three were Omps, 13 were Imps, eight were Pps and five were Cps. Additional file 2 summarizes findings for genes that were downregulated under mimetic conditions with a log2 > 10.

### Gene ontology (GO) term enrichment analysis

Among the genes upregulated under mimetic conditions, only the GO term GO:0003676 (nucleic acid binding) was found to be enriched and associated with the presence in 37 upregulated genes (Table 3). With regard to genes downregulated under mimetic conditions, 31 GO terms were classified as being enriched (Table 4).

**Table 3:** List of upregulated genes where GO term GO:0003676 (nucleic acid binding) was found

*Locus*	GenBank product	*Locus*	GenBank product
*HAPS_RS06635*	tRNA-dihydrouridine synthase	*HAPS_RS06640*	Fis family transcriptional regulator
*HAPS_RS07775*	30S ribosomal protein S10	*HAPS_RS07795*	50S ribosomal protein L2
*HAPS_RS01040*	LysR family transcriptional regulator	*HAPS_RS07785*	50S ribosomal protein L4
*HAPS_RS03460*	tRNA pseudouridine (65) synthase TruC	*HAPS_RS01015*	translation initiation factor IF-2
*HAPS_RS07435*	MurR/RpiR family transcriptional regulator	*HAPS_RS07790*	50S ribosomal protein L23
*HAPS_RS02800*	transcriptional regulator	*HAPS_RS10775*	ATPase AAA
*HAPS_RS02140*	transcriptional regulator	*HAPS_RS06525*	Holliday junction DNA helicase RuvA
*HAPS_RS09580*	23S rRNA (guanosine-2’-O-)-methyltransferase	*HAPS_RS07720*	RNA polymerase, beta subunit
*HAPS_RS08560*	restriction endonuclease	*HAPS_RS01010*	transcription termination factor NusA
*HAPS_RS09560*	30S ribosomal protein S7	*HAPS_RS07780*	50S ribosomal protein L3
*HAPS_RS09565*	elongation factor G	*HAPS_RS01395*	elongation factor Ts
*HAPS_RS08275*	endonuclease	*HAPS_RS02855*	single-stranded DNA-binding protein
*HAPS_RS10985*	Fur family transcriptional regulator	*HAPS_RS00670*	transposase
*HAPS_RS02340*	RNA helicase	*HAPS_RS10560*	transcriptional regulator
*HAPS_RS04055*	DNA polymerase III subunit epsilon	*HAPS_RS03180*	50S ribosomal protein L25
*HAPS_RS01380*	lysine tRNA synthetase	*HAPS_RS04045*	heat-shock protein
*HAPS_RS08565*	restriction endonuclease	*HAPS_RS01070*	tRNA s (4) U8 sulfurtransferase
*HAPS_RS08440*	transcriptional regulator	*HAPS_RS04180*	formamidopyrimidine-DNA glycosylase
*HAPS_RS05455*	DNA-binding protein		

The locus in the reference genome (SH0165 strain) and GenBank product are indicated

**Table 4:** GO terms found to be enriched among downregulated genes under mimetic conditions

GO term	GO domain	Number of genes
GO:0008643 *carbohydrate transport*	BP	16
GO:0015980 *energy derivation by oxidation of organic compounds*	BP	17
GO:0055114 *oxidation-reduction process*	BP	21
GO:0009401 *phosphoenolpyruvate-dependent sugar phosphotransferase system*	BP	13
GO:0009401 *phosphoenolpyruvate-dependent sugar phosphotransferase system*	BP	13
GO:0006091 *generation of precursor metabolites and energy*	BP	19
GO:0045333 *cellular respiration*	BP	14
GO:0006099 *tricarboxylic acid cycle*	BP	10
GO:0006099 *tricarboxylic acid cycle*	BP	10
GO:0009060 *aerobic respiration*	BP	10
GO:0044712 *single-organism catabolic process*	BP	17
GO:0044699 *single-organism process*	BP	73
GO:0005975 *carbohydrate metabolic proces*s	BP	25
GO:0044724 *single-organism carbohydrate catabolic process*	BP	11
GO:0044282 *small molecule catabolic process*	BP	12
GO:0016052 *carbohydrate catabolic process*	BP	11
GO:1901575 *organic substance catabolic process*	BP	18
GO:1901476 *carbohydrate transporter activity*	MF	8
GO:0015144 *carbohydrate transmembrane transporter activity*	MF	8
GO:0044765 *single-organism transport*	BP	23
GO:0009056 *catabolic process*	BP	18
GO:0005996 *monosaccharide metabolic process*	BP	10
GO:1902578 *single-organism localization*	BP	23
GO:0071702 *organic substance transport*	BP	17
GO:0044723 *single-organism carbohydrate metabolic process*	BP	17
GO:0006810 *transport*	BP	26
GO:0019318 *hexose metabolic process*	BP	8
GO:0051179 *localization*	BP	26
GO:0051234 *establishment of localization*	BP	26
GO:0044710 *single-organism metabolic process*	BP	49
GO:0046365 *monosaccharide catabolic process*	BP	5

The number of genes where each GO term was found is indicated. The gene ontology domain (GO domain) to which each GO term belongs is also indicated, *BP* biological process, *MF* molecular function

## Discussion

Most bacteria reshape their coating structures inside the host since they need to adapt to a new potentially harmful environment [6]. Although the environment that a microorganism endures inside the host is much more complex than that replicated in the laboratory, the two selected conditions in this study (iron-restriction and temperature higher than 37 °C) attempted to partially simulate the infection in natural conditions. Some reports have been carried out on the changes occurring in the *H.* (*G.*) *parasuis* transcriptome when this bacterium was subjected to these two mimetic conditions of high temperature and iron scarcity [10, 12, 23]. However, the two circumstances in those studies were not tested together because these studies were focused in the understanding of both metabolism and virulence factors but not to search putative candidates that could be used as vaccine antigens.

The bacterial mechanisms used to remove iron from the host need surface-exposed proteins [9], and their expression is induced by a low iron concentration [24]. Among the genes upregulated under mimetic conditions, we detected six genes coding for Eps or Omps related to the obtaining of iron from the host (TbpA, TbpB, HxuA, HxuB, HxuC and FhuA). In a previous study of the transcriptome of *H. (G.) parasuis* exposed to the intraalveolar environment for two hours, upregulation of genes encoding membrane proteins involved in iron uptake were detected [25]. This finding reinforces our results concerning the high probability that the six above mentioned genes will be overexpressed during infection with *H. (G.) parasuis*.

TbpA and TbpB are porcine transferrin binding proteins that show different protection degrees against Glässer’s disease [26, 27]. HxuA, HxuB and HxuC correspond to hemophore, transporter and receptor of the heme/hemopexin-binding protein (hxu) operon, respectively [28]. A protection of 87.5% for HxuC, 62.5% for HxuB and 37.5% for HxuA has been recently showed in mice against *H. (G.) parasuis* [29]. The upregulated gene *HAPS_RS00485*, coding for FhuA protein, a receptor for siderophores, has not been tested as vaccine antigen until date [30]. Curiously, Melnikow et al. [10] did not find *fhuA* among the genes upregulated under iron-restrictive conditions. One possible explanation for the difference between this study and ours could be that the combination of both conditions is required for upregulation of this gene.

Depending on the bacterial species, fever could cause a heat stress [31] that triggered changes involving coating structures [6]. It would be therefore expected that the reprogramming of the transcriptome undergone by the bacterium to resist heat stress could affect some genes encoding for surface-exposed proteins. The upregulated gene *tolC* found in this study encodes an Omp (TolC) lipoprotein that forms a trimeric channel and acts in the transport of several molecules [32]. Li et al. [33] observed that mice immunized with this protein and then challenged with *H.* (*G.*) *parasuis* showed a survival rate of 80%.

In addition to these well-characterized proteins in the databases, another five proteins were predicted to be located on the bacterial surface (Eps or Omps). The largest of them was an autotransporter with a serine protease domain which is annotated as two different genes (*HAPS_RS00740* and *HAPS_RS00745*) in the reference genome (SH0165 strain) because it presents a punctual mutation that triggers off the appearance of a stop codon. This long protein presents a broad sequence homology with the AasP autotransporter of *A. pleuropneumoniae*, which it is upregulated when this bacterium is grown under iron restriction stress [34]. On the other hand, a previous study performed with an *AasP* mutant *in A. pleuropneumoniae* showed that AasP protein is involved in adhesion under iron-restriction conditions [35].

The upregulated gene *HAPS_RS04485* codes a protein that harbors domains with an immunoglobulin-like fold, which are usually present in proteins related to invasion or adhesion in prokaryotes [36]. Among the three remaining surface proteins whose genes were upregulated, two of them (*HAPS_RS00735* and *HAPS_RS01895*) neither presented homology to known proteins nor harbored domains with a known function, while in the remaining one (*HAPS_RS10780*) only a β-barrel and a single tetratricopeptide repeat could be detected.

With regard to the functional enrichment analysis under mimetic conditions to natural infection, only one significant enrichment was observed in the GO term 0003676, corresponding to nucleic acid binding, which was found in 37 upregulated genes. This could be consistent with the important reprogramming, at both transcriptional and translational levels, which the bacteria undergo when exposed to environmental stress [37]. Among these genes, six coded ribosomal subunit binding proteins, four coded rRNA-related enzymes (translational level) and eight coded transcriptional regulators (transcriptional level). Within the upregulated transcriptional regulators, the iron uptake regulatory protein (Fur) must be highlighted. This protein forms a dimer in the presence of Fe^2+^ that represses the expression of genes related to iron uptake. However, when available iron is scarce, this dimer breaks down and transcription of genes related to iron uptake is allowed [38]. The upregulation of a negative regulator of iron acquisition under iron-restricted conditions may be paradoxical effect; however, different findings have been shown over the last years that the role of Fur is not as simple as it had been previously stated although it remains fully valid. There are positively regulated genes for Fe-induced Fur dimers and other positively or negatively regulated genes for Fur when this protein does not form a complex with Fe^2+^. Some of the genes that are regulated in these ways are related to the response to stressful conditions or to virulence [38]. Therefore, it was not unusual to find *Fur* gene among those upregulated under our stress conditions.

Concerning functional enrichment analysis of downregulated genes under mimetic conditions, the most enriched terms were related to energy metabolism, redox reactions, or to both of them. Relative to energy metabolism, this finding would make sense with slowing of growth braking expected in a stressful environment, which should result in a general decrease in the metabolism. In the case of redox processes, it might simply be a reflection of the decrease in bacterial growth and/or it could be that the microorganism, when detecting iron deficiency, prefers not to waste energy in the production of compounds that cannot perform their function since the iron is the main limiting factor [9].

As previously mentioned, microorganisms have to face a number of stressors during infection, such as unfavorable temperatures, pH changes or free radicals, which trigger the expression of several proteins known as cellular stress proteins [39]. These proteins behave as chaperones promoting the assembly of other macromolecules and are often called heat shock proteins (Hsp) since it was previously believed that they acted alone against heat stress [40]. Several genes encoding different Hsp were upregulated in our study, such as DnaJ, DnaK, HslO and HslR. Several Hsp behaving as surface antigens were observed, which might seem a contradiction considering that the role played as chaperones should imply an intracellular location. However, many of these proteins, in addition to act as chaperones, also present functions related to pathogenesis that may imply a superficial location [40]. Accordingly, it would be reasonable to study these proteins as vaccine antigens; however, it would not be appropriate to do because Hsp have been highly conserved throughout evolution and there is a high homology between the Hsp from bacterial and those from mammalian origin. For this reason, the use of Hsp as vaccine antigens could trigger autoimmune diseases [39].

## Conclusion

Slowing of growth expected in stressful conditions have given rise to the upregulation of 13 *H.* (*G.*) *parasuis* genes coding for proteins located on the bacterial surface. Among them, seven proteins untested to date were detected as vaccine antigens: FhuA (encoded by *HAPS_RS00485*), a fimbrial usher protein (encoded by *HAPS_RS03735*), a long autotransporter (encoded by *HAPS_RS00740* and *HAPS_RS00745*), a protein containing domains with an Ig-like fold (encoded by *HAPS_RS04485*) and other three surface proteins without known function (encoded by *HAPS_RS10780*, *HAPS_RS00735* and *HAPS_RS01895*). These seven novel vaccine candidates could provide protection against Glässer disease, but their effectiveness have to be tried in future studies. Anyway, their expression must be sustained in other serovars different from serovar 5.

## Methods

### Bacterial strain and growth conditions

*H.* (*G.*) *parasuis* was grown under (i) in vitro optimal culture conditions (control conditions) and (ii) under in vitro growth conditions partially mimicking the host environment encountered during infection (iron-restriction and temperature stress by raising incubation temperature above 37 °C). The transcriptomes from both growths were compared by RNA sequencing to detect overexpressed genes coding proteins exposed on the bacterial surface.

For control culture conditions (without iron-restriction and 37 °C), *H.* (*G.*) *parasuis* Nagasaki strain (reference strain of serovar 5 kindly supplied by Kielstein P., Federal Institute for Health Protection of Consumers and Veterinary Medicine, Jena, Germany) was inoculated into 30 ml of PPLO broth (Conda Laboratories, Spain) with 150 μM nicotinamide adenine dinucleotide (NAD, Sigma-Aldrich, Spain) and 0.075% glucose (Sigma-Aldrich), and it was cultured at 37 °C until reaching an optical density of 0.5 at 600 nm (OD_600_). Three replicates were made under these conditions. For mimetic conditions (iron-restriction and 41 °C), *H.* (*G.*) *parasuis* Nagasaki strain was inoculated into 30 ml of the same broth and was cultured at 41 °C until reaching an OD_600_ of 0.3. Then, iron was restricted by adding 200 μM 2 2′-dipyridyl, and the culture was grown again at 41 °C to an OD_600_ of 0.5. Three replicates were also made.

### RNA extraction and sample preparation

When the appropriate OD_600_ was reached for each replicate, the culture was centrifuged at 7000×*g* and 4 °C for 7 min. The supernatants were removed and the pellets were preserved on ice. RNA extraction was performed using the High Pure RNA Isolation kit (Sigma-Aldrich) following the manufacturer’s specifications. The DNA-free kit (Thermo-Fisher) was used to remove the contaminating DNA, which was verified by species-specific PCR [13], testing the absence of amplification in samples treated with DNAse. Finally, RNA concentrations were measured in a NanoDrop 1000 (Thermo-Fisher), and samples were stored at − 80 °C. The RNA integrity checking was tested using a Bioanalyzer Agilent 2100 (Agilent Technologies, Spain) from the Laboratory of Instrumental Techniques (University of León, Spain).

### Library preparation and Illumina sequencing

Ribosomal RNA (rRNA) was removed from the samples using a Ribo-Zero Magnetic Kit Bacteria (Illuminia, Portugal). Libraries were then prepared following the instructions of the NEBNext Ultra Directional RNA Library Prep kit for Illumina (New England Biolabs, USA). The input of ribosome-depleted RNA to start the protocol was 10 ng quantified by an Agilent 2100 Bioanalyzer using a RNA 6000 nano LabChip kit (Agilent Technologies, Germany). The fragmentation time used was 15 min. The cDNA libraries obtained were validated and quantified by an Agilent 2100 Bioanalyzer using a DNA7500 LabChip kit (Agilent Technologies). An equimolecular pool of libraries were titrated by quantitative PCR using the Kapa-SYBR FAST qPCR kit for LightCycler480 (Kapa BioSystems, USA), and a reference standard for quantification. The pool of libraries was denatured prior to be seeded on a flowcell at a density of 2 2 pM, where clusters were formed and sequenced with a depth of 10 M using a NextSeq 500 High Output Kit (Illumin) in a 1 × 75 single-read sequencing run on a NextSeq 500 sequencer (Illumin).

### Bioinformatic analysis of differential gene expression

Bioinformatic analysis for the RNAseq data was performed by Era7 bioinformatics (Spain) following the protocol described by Trapnell et al. [14]. Quality control of raw readings was performed with the FastQC tool (http://www.bioinformatics.babraham.ac.uk/projects/fastqc/). Alignment to the reference genome was performed using TopHat2 software, but since the genome of the Nagasaki strain has not been completely sequenced, the SH0165 strain genome (GenBank accession nr NC_011852.1), also belonging to serovar 5, was used as the reference genome. Transcripts assembly was performed with the Cufflinks tool and transcripts merge with the Cuffmerge tool. Analysis of differences in gene expression was performed with Cuffdiff. Statistical significance was considered for *p* < 0.05 (corrected by the multiple testing Benjamini-Hochberg method). Only genes with a log_2_ (fold change) being > 10 were considered for further analyses.

### Studies of differentially expressed genes

Differentially expressed genes were subjected to search in the GenBank database to discard pseudogenes and tRNA genes. GenBank [15] and Uniprot [16] annotation was obtained to genes and proteins differentially expressed. The sequences of the proteins encoded by differentially expressed genes were obtained using QuickGo [17]. Prediction of the protein cellular location was carried out using the CELLO v 2.5 web tool (http://cello.life.nctu.edu.tw/) [18]. Proteins predicted to be related to pathogenesis were searched for using the MP3 web tool (http://metagenomics.iiserb.ac.in/mp3/index.php) [19]. Proteins that were not identified in the GenBank or Uniprot databases but were considered of interest (predicted to be found on the bacterial surface or related to pathogenesis) were subjected to a study using BLASTp in order to find orthologous proteins in related species and using GenBank or InterproScan databases [20] to find domains of a known function. Databases and BLASTp were also used to verify the protein location that CELLO assigned as belonging to the extracellular and Omp fractions.

Genes upregulated under culture mimetic conditions are referred to as “upregulated under mimetic conditions”, while those genes upregulated under control conditions are referred to as “downregulated under mimetic conditions”. Only genes with log_2_ (fold change) was > 10 blank were considered in the analysis of data.

### Gene ontology (GO) term enrichment analysis

Functional enrichment analysis in terms of gene ontology (GO) of differentially expressed genes was conducted using the DAVID v 6.7 web server (https://david.ncifcrf.gov/home.jsp) [21]. Only the GO terms containing a minimum of five differentially expressed genes were considered and the GO terms that showed a *p* < 0.05 (corrected by the multiple testing Benjamini-Hochberg method) were considered significantly enriched.

## Additional files

Additional file 1: Summary of genes that were upregulated under mimetic conditions with a log_2_ (fold change) > 10. Indicated findings in GenBank and Uniprot databases of genes that were upregulated under mimetic conditions with a log_2_ (fold change) > 10. P indicates that the encoded protein is related to pathogenesis, and NP indicates that there is no relationship with pathogenesis. The location of the protein is indicated with EX (extracellular), OM (outer membrane), PP (periplasmic), IM (inner membrane) or CP (cytoplasmic). * indicates that they are the same protein (PDF 117 kb).

Additional file 2: Summary of genes that were downregulated under mimetic conditions with a log_2_ (fold change) > 10. Indicated findings in GenBank and Uniprot databases of genes that were downregulated under mimetic conditions with a log_2_ (fold change) > 10. P indicates that the encoded protein is related to pathogenesis, and NP indicates that there is no relationship with pathogenesis. The location of the protein is indicated with EX (extracellular), OM (outer membrane), PP (periplasmic), IM (inner membrane) or CP (cytoplasmic). * indicates that they are the same protein (PDF 127 kb).

## Abbreviations

Cps: Cytoplasmic proteins
Eps: Extracellular proteins
Fur: Ferric uptake regulatory protein
GO: Gene ontology
Hsp: Heat shock proteins
Imps: Inner membrane proteins
Omps: Outer membrane proteins
Pps: Periplasmic proteins
RNAseq: RNA-sequencing
