# Supplementary material for: Transcriptomics of Haemophilus (Glässerella) parasuis serovar 5 subjected to culture conditions partially mimetic to natural infection for the search of new vaccine antigens
Source: BMC Vet Res. 2018 Nov 6;14:326. doi: 10.1186/s12917-018-1647-1 (PMC6219065; doi:10.1186/s12917-018-1647-1)
Supplement: Supplementary file 1 — Summary of genes that were upregulated under mimetic conditions with a log2 (fold change) > 10. Indicated findings in GenBank and Uniprot databases of genes that were upregulated under mimetic conditions with a log2 (fold change) > 10. P indicates that the encoded protein is related to pathogenesis, and NP indicates that there is no relationship with pathogenesis. The location of the protein is indicated with EX (extracellular), OM (outer membrane), PP (periplasmic), IM (inner membrane) or CP (cytoplasmic). * indicates that they are the same protein (PDF 117 kb). [file 12917_2018_1647_MOESM1_ESM.pdf]

Locus	**Genbank product**	**no. Uniprot**	**Name Uniprot**	**Pathogeny**	**Localization**
HAPS_RS00115	plasmid stability protein StbD	-	-	NP	CP
HAPS_RS00120	RelE toxin	B8F327	|stbE|Putative plasmid stability protein StbE, addiction module antitoxin	NP	CP
HAPS_RS00325	GTP-binding protein	B8F366	|typA|GTP-binding protein/membrane GTPase involved in stress response	NP	CP
HAPS_RS00370	protein TolA	B8F375	|tolA|Cell envelope integrity inner membrane protein TolA	P	IM
HAPS_RS00425	sugar ABC transporter substrate-binding protein	B8F386	|lptA|Lipopolysaccharide export system protein LptA	NP	PP
HAPS_RS00480	pseudo	-	-	-	-
HAPS_RS00485	TonB-dependent receptor	-	-	P	OM
HAPS_RS00635	hypothetical protein	-	-	NP	CP
HAPS_RS00670	transposase	B8F3D0	|HAPS_0133|Uncharacterized protein	NP	PP
HAPS_RS00730	acetolactate synthase	B8F3E1	|ilvM|Acetolactate synthase 2 regulatory subunit	NP	CP
HAPS_RS00735	hypothetical protein	-	-	P	OM
HAPS_RS00740*	autotransporter domain-containing protein*	-	-	P*	OM*
HAPS_RS00745*	hypothetical protein*	-	-	P*	EX*
HAPS_RS00825	hypothetical protein	-	-	NP	CP
HAPS_RS01010	transcription termination factor NusA	B8F3J4	|nusA|Transcription termination/antitermination protein NusA	NP	CP
HAPS_RS01015	translation initiation factor IF-2	B8F3J5	|infB|Translation initiation factor IF-2	NP	CP
HAPS_RS01040	LysR family transcriptional regulator	B8F3J8	|HAPS_0207|Putative HTH-type transcriptional regulator	NP	CP
HAPS_RS01065	pseudo	-	-	-	-
HAPS_RS01070	tRNA s(4)U8 sulfurtransferase	B8F3K3	|thiI|tRNA sulfurtransferase	NP	CP
HAPS_RS01075	bifunctional N-acetylglucosamine-1-phosphate uridyltransferase/glucosamine-1-phosphate acetyltransferase	B8F3K4	|glmU|Bifunctional protein GlmU	NP	CP
HAPS_RS01255	ABC transporter permease	B8F3P0	|HAPS_0253|ABC-type nitrate/sulfonate/bicarbonate transport permease	P	IM
HAPS_RS01260	ABC transporter substrate-binding protein	B8F3P1	|HAPS_0254|ABC-type nitrate/sulfonate/bicarbonate transport systems periplasmic components protein	P	CP
HAPS_RS01265	ABC transporter ATP-binding protein	B8F3P2	|HAPS_0255|ABC-type nitrate/sulfonate/bicarbonate transport system, ATPase	P	IM
HAPS_RS01270	acyl-CoA dehydrogenase	B8F3P3	|HAPS_0256|Acyl-CoA dehydrogenase	NP	CP
HAPS_RS01380	lysine tRNA synthetase	B8F3R5	|lysS|Lysine--tRNA ligase	NP	CP
HAPS_RS01385	peroxide stress protein YaaA	B8F3R6	|HAPS_0280|UPF0246 protein HAPS_0280	NP	CP
HAPS_RS01395	elongation factor Ts	B8F3R8	|tsf|Elongation factor Ts	NP	CP
HAPS_RS01400	sulfurtransferase FdhD	-	-	P	CP
HAPS_RS01430	pseudo	-	-		
HAPS_RS01435	hypothetical protein	B8F3S4	|HAPS_0289|Uncharacterized protein	P	CP
HAPS_RS01560	tRNA 2-thiocytidine biosynthesis protein TtcA	-	-	NP	CP
HAPS_RS01750	hypothetical protein	-	-	NP	CP

Locus	Genbank product	no. Uniprot	Name Uniprot	Pathogeny	Localization
HAPS_RS01805	ABC transporter ATPase	B8F3Z5	|HAPS_0364|ATPase components of ABC transporters with duplicated ATPase domains-containing protein	P	PP
HAPS_RS01810	iron transporter	B8F3Z6	|fieF|Cation-efflux pump FieF, predicted Co/Zn/Cd cation transporter	NP	IM
HAPS_RS01830	hypothetical protein	-	-	NP	CP
HAPS_RS01835	hypothetical protein	B8F3Z9	|HAPS_0370|Uncharacterized protein	NP	CP
HAPS_RS01895	hypothetical protein	B8F410	|HAPS_0382|Uncharacterized protein	P	EX
HAPS_RS01900	esterase	B8F411	|mhpC|Putative esterase	NP	PP
HAPS_RS02075	pseudo	-	-		
HAPS_RS02080	ATPase	-	-	NP	CP
HAPS_RS02140	transcriptional regulator	B8F450	|HAPS_0433|Predicted transcription regulator containing HTH domain OS=Haemophilus parasuis serovar 5 (strain SH0165) GN=HAPS_0433 PE=4 SV=1	NP	CP
HAPS_RS02145	toxin RelE	B8F451	|HAPS_0434|Putative membrane protein	NP	CP
HAPS_RS02340	RNA helicase	B8F490	|deaD|ATP-dependent RNA helicase DeaD	NP	CP
HAPS_RS02480	Nif3-like dinuclear metal center hexameric protein	B8F4B4	|HAPS_0502|GTP cyclohydrolase 1 type 2 homolog	NP	CP
HAPS_RS02610	hypothetical protein	B8F4D5	|purL|Phosphoribosylformylglycinamidine synthase	P	CP
HAPS_RS02650	hypothetical protein	-	-	NP	CP
HAPS_RS02685	molecular chaperone DnaK	B8F4E6	|dnaK|DnaK suppressor protein/ C4-type zinc finger protein, DksA/TraR family	NP	CP
HAPS_RS02690	bacteriophage P2 Tail completion protein GPR	B8F4E7	|HAPS_0565|Putative bacteriophage protein, putative transcription regulator, cro/C1-type DNA-binding domain	NP	CP
HAPS_RS02695	virion morphogenesis protein	B8F4E8	|gpS|Bacteriophage tail completion protein gpS-like protein/phage virion morphogenesis protein	NP	CP
HAPS_RS02800	transcriptional regulator	B8F4G7	|HAPS_0565|Putative bacteriophage protein, putative transcription regulator	NP	CP
HAPS_RS02830	hypothetical protein	B8F4H6	|HAPS_0575|Uncharacterized protein	NP	CP
HAPS_RS02850	translocation protein TolB precursor	-	-	NP	CP
HAPS_RS02855	single-stranded DNA-binding protein	B8F4I1	|ssb|Single-stranded DNA-binding protein	NP	PP
HAPS_RS02955	hypothetical protein	B8F4K1	|HAPS_0600|Phage protein	NP	CP
HAPS_RS03100	hypothetical protein	B8F4N2	|HAPS_0631|Uncharacterized protein	NP	CP
HAPS_RS03150	tRNA-Val	-	-		
HAPS_RS03180	50S ribosomal protein L25	B8F4P6	|rplY|50S ribosomal protein L25	NP	CP
HAPS_RS03215	potassium transporter	B8F4Q4	|kefBC|Glutathione-regulated potassium-efflux system protein	NP	IM
HAPS_RS03450	N-acetylmuramic acid 6-phosphate etherase	B8F4V1	|murQ|N-acetylmuramic acid 6-phosphate etherase	NP	CP
HAPS_RS03455	anhydro-N-acetylmuramic acid kinase	B8F4V2	|HAPS_0708|Uncharacterized protein	NP	CP
HAPS_RS03460	tRNA pseudouridine(65) synthase TruC	B8F4V3	|truC|tRNA pseudouridine synthase C, pseudouridylate synthase, 23S RNA-specific	NP	CP
HAPS_RS03710	hypothetical protein	-	-	NP	CP
HAPS_RS03735	fimbrial usher protein	-	-	P	OM
HAPS_RS03755	predicted ATPase involved in chromosome partitioning	-	-	NP	CP

Locus	Genbank product	no. Uniprot	Name Uniprot	Pathogeny	Localization
HAPS_RS03770	hypothetical protein	B8F511	|HAPS_0775|Putative prophage primase	NP	CP
HAPS_RS03820	tRNA-Leu	-	-	-	-
HAPS_RS04045	heat-shock protein	B8F560	|hslR|Heat shock-like protein 15	NP	CP
HAPS_RS04050	redox-regulated molecular chaperone Hsp33	B8F561	|hslO|33 kDa chaperonin	NP	CP
HAPS_RS04055	DNA polymerase III subunit epsilon	B8F562	|dnaQ|DNA polymerase III subunit epsilon	NP	CP
HAPS_RS04100	peptide chain release factor 2	-	-	NP	CP
HAPS_RS04180	formamidopyrimidine-DNA glycosylase	B8F586	|mutM|Formamidopyrimidine-DNA glycosylase	NP	CP
HAPS_RS04480	hypothetical protein	-	-	P	PP
HAPS_RS04485	hypothetical protein	B8F5E9	|HAPS_0923|Uncharacterized protein	P	EX
HAPS_RS04635	threonyl-tRNA synthetase	B8F5H9	|thrS|Threonine--tRNA ligase	NP	CP
HAPS_RS04900	transporter	B8F5M4	|alsT|Na+/alanine symporter	NP	IM
HAPS_RS05120	heme utilization protein HuvX	-	-	NP	CP
HAPS_RS05125	heme utilization protein HutZ	B8F5S2	|hugZ|Heme utilization protein	NP	CP
HAPS_RS05320	ribosome small subunit-dependent GTPase	-	-	NP	CP
HAPS_RS05350	pseudo	-	-	-	-
HAPS_RS05455	DNA-binding protein	B8F5Y2	|hns|DNA-binding protein	NP	CP
HAPS_RS05485	ABC transporter ATP-binding protein	-	-	NP	CP
HAPS_RS05580	antirepressor	B8F607	|HAPS_1148|Possible prophage antirepressor	NP	CP
HAPS_RS05800	pseudo	-	-	-	-
HAPS_RS05955	arginyl-tRNA synthetase	-	-	NP	CP
HAPS_RS05960	pseudo	-	-	-	-
HAPS_RS06085	membrane protein	B8F693	|accD|Acetyl-CoA carboxylase beta subunit	NP	CP
HAPS_RS06380	glycerol-3-phosphate dehydrogenase subunit C	B8F6F2	|glpC|sn-glycerol-3-phosphate dehydrogenase subunit C	NP	CP
HAPS_RS06515	putrescine/spermidine ABC transporter substrate-binding protein	B8F6H2	|potD|Putrescine-binding periplasmic protein	NP	PP
HAPS_RS06520	hypothetical protein	B8F6H3	|yaaH|Permease, Inner membrane protein yaaH	P	IM
HAPS_RS06525	Holliday junction DNA helicase RuvA	-	-	NP	CP
HAPS_RS06610	cell envelope protein TonB	B8F6J2	|tonB|Protein TonB	P	PP
HAPS_RS06615	TonB system transport protein ExbD	B8F6J3	|exbD|TonB system transport protein ExbD type-2/biopolymer transport protein	NP	CP
HAPS_RS06635	tRNA-dihydrouridine synthase	B8F6J7	|dusB|tRNA-dihydrouridine synthase B	NP	CP
HAPS_RS06640	Fis family transcriptional regulator	B8F6J8	|fis|DNA-binding protein Fis	P	PP
HAPS_RS06835	4'-phosphopantetheinyl transferase	B8F6M9	|yieE|Phosphopantetheinyl transferase	NP	CP
HAPS_RS06865	hypothetical protein	B8F6N5	|HAPS_1415|Uncharacterized protein	NP	CP
HAPS_RS06890	protease	B8F6P0	|prtC|Collagenase prtC/related protease involved in cellular process and singaling	NP	CP
HAPS_RS07435	MurR/RpiR family transcriptional regulator	B8F6Y4	|rpiR|RpiR family transcriptional regulator	NP	CP
HAPS_RS07625	heme ABC transporter ATP-binding protein	B8F722	|hmuV|Hemin transport system ATP-binding protein HmuV	NP	CP
HAPS_RS07630	iron ABC transporter permease	B8F723	|hmuU|Hemin transport system permease protein HmuU	P	IM

Locus	Genbank product	no. Uniprot	Name Uniprot	Pathogeny	Localization
HAPS_RS07635	hemin ABC transporter substrate-binding protein	-	-	NP	IM
HAPS_RS07720	RNA polymerase, beta subunit	B8F741	|rpoB|DNA-directed RNA polymerase subunit beta	NP	CP
HAPS_RS07775	30S ribosomal protein S10	B8F754	|rpsJ|30S ribosomal protein S10	NP	CP
HAPS_RS07780	50S ribosomal protein L3	B8F755	|rplC|50S ribosomal protein L3	NP	CP
HAPS_RS07785	50S ribosomal protein L4	B8F756	|rplD|50S ribosomal protein L4	NP	CP
HAPS_RS07790	50S ribosomal protein L23	B8F757	|rplW|50S ribosomal protein L23	NP	CP
HAPS_RS07795	50S ribosomal protein L2	B8F758	|rplB|50S ribosomal protein L2	NP	CP
HAPS_RS07950	hypothetical protein	-	-	P	CP
HAPS_RS07955	membrane protein	B8F786	|pykF|Pyruvate kinase	NP	PP
HAPS_RS08275	endonuclease	B8F7D1	|nfo|Probable endonuclease 4	NP	CP
HAPS_RS08315	hypothetical protein	B8F7D5	|HAPS_1712|Uncharacterized protein	NP	IM
HAPS_RS08435	addiction module protein	B8F7F5	|hicA|Addiction module killer protein	NP	CP
HAPS_RS08440	transcriptional regulator	B8F7F6	|HAPS_1738|Possible transcriptional regulator	NP	CP
HAPS_RS08560	restriction endonuclease	B8F7H2	|bcgIA|Restriction enzyme, alpha subunit/N-6 DNA methylase	NP	CP
HAPS_RS08565	restriction endonuclease	B8F7H3	|bcgIB|Restriction enzyme, beta subunit/N-6 DNA methylase	NP	CP
HAPS_RS08570	restriction endonuclease	-	-	NP	CP
HAPS_RS08685	hypothetical protein	-	-	NP	CP
HAPS_RS09000	hypothetical protein	B8F7Q0	|HAPS_1850|Uncharacterized protein	P	IM
HAPS_RS09165	molecular chaperone DnaJ	B8F7S3	|dnaJ|Chaperone protein DnaJ	NP	CP
HAPS_RS09170	virulence-associated protein VapD	B8F7S4	|vapD|Virulence-associated protein D	NP	CP
HAPS_RS09385	spermidine/putrescine ABC transporter substrate-binding protein	-	-	NP	PP
HAPS_RS09560	30S ribosomal protein S7	B8F7Z3	|rpsG|30S ribosomal protein S7	NP	CP
HAPS_RS09565	elongation factor G	B8F7Z4	|fusA|Elongation factor G	NP	CP
HAPS_RS09580	23S rRNA (guanosine-2'-O-)-methyltransferase	B8F7Z6	|rlmB|23S rRNA (guanosine-2'-O-)-methyltransferase RlmB	NP	CP
HAPS_RS09625	transposase	-	-	NP	CP
HAPS_RS10065	C4-dicarboxylate ABC transporter	B8F879	|dcuB|Anaerobic C4-dicarboxylate transporter	NP	IM
HAPS_RS10195	*DUF262 domain-containing protein*	B8F899	|HAPS_2100|Uncharacterized protein	P	CP
HAPS_RS10260	dUMP phosphatase	B8F8A7	|HAPS_2113|Nucleotidase/putative HAD superfamily haloacid dehalogenase-like hydrolase	NP	CP
HAPS_RS10530	TolC family protein	B8F8F2	|tolC|RND efflux system outer membrane lipoprotein/RND superfamily resistance-nodulation-cell division antiporter	P	OM
HAPS_RS10560	transcriptional regulator	B8F8F7	|HAPS_2174|XRE family transcriptional regulator	NP	CP
HAPS_RS10565	hypothetical protein	B8F8F8	|HAPS_2175|Uncharacterized protein	NP	CP
HAPS_RS10585	ligand-gated channel	B8F8G2	|hxuC|Heme/hemopexin utilization protein C/outer membrane receptor protein, mostly Fe transport	P	OM
HAPS_RS10590	ShlB/FhaC/HecB family hemolysin secretion/activation protein	B8F8G3	|hxuB|Heme/hemopexin-binding protein B, hemolysin activation/secretion protein	P	OM

Locus	Genbank product	no. Uniprot	Name Uniprot	Pathogeny	Localization
HAPS_RS10595	hypothetical protein	B8F8G4	|hxuA|Heme/hemopexin-binding protein A (Heme:hemopexin utilization protein A)	P	EX
HAPS_RS10730	glycosyl transferase	B8F8I8	|lsgE|Putative lipooligosaccharide galactosyltransferase involved in cell wall biogenesis	NP	CP
HAPS_RS10735	amylovoran biosynthesis protein AmsE	B8F8I9	|lsgF|Putative UDP-galactose--lipooligosaccharide galactosyltransferase	NP	CP
HAPS_RS10765	rod shape-determining protein MreC	-	-	NP	IM
HAPS_RS10770	rod shape-determining protein MreD	B8F8J7	|mreD|Rod shape-determining protein MreD	NP	IM
HAPS_RS10775	ATPase AAA	B8F8J8	|rarA|Recombination factor protein RarA	NP	CP
HAPS_RS10780	membrane protein	B8F8J9	|HAPS_2219|Possible outer membrane protein/FOG: TPR repeat protein	P	OM
HAPS_RS10785	hypothetical protein	-	-	NP	PP
HAPS_RS10790	biopolymer transporter ExbB	B8F8K1	|exbB|Biopolymer transport ExbB protein	NP	IM
HAPS_RS10795	biopolymer transporter ExbD	-	-	NP	CP
HAPS_RS10800	transferrin-binding protein-like solute binding protein	-	-	P	OM
HAPS_RS10805	lactoferrin/transferrin family TonB-dependent receptor	B8F8K4	|tbpA|Transferrin-binding protein 1	P	OM
HAPS_RS10825	non-canonical purine NTP pyrophosphatase RdgB/HAM1 family	-	-	NP	CP
HAPS_RS10830	deoxyribonucleotide triphosphate pyrophosphatase	B8F8K9	|HAPS_2229|Uncharacterized protein	NP	CP
HAPS_RS10835	YcgN family cysteine cluster protein	B8F8L0	|HAPS_2230|Uncharacterized protein	NP	CP
HAPS_RS10980	flavodoxin	B8F8N8	|fldA|Flavodoxin	NP	CP
HAPS_RS10985	Fur family transcriptional regulator	B8F8N9	|fur|Ferric uptake regulation protein	NP	CP
HAPS_RS11010	transpeptidase	B8F8P5	|HAPS_2267|Uncharacterized protein	P	PP
HAPS_RS11030	transcriptional regulator	B8F8P9	|impA|SOS-response transcriptional repressor	P	CP
HAPS_RS11145	pseudo	-	-	-	-
HAPS_RS11150	acyl-CoA esterase	B8F8S2	|HAPS_2296|Uncharacterized protein	NP	CP
