# Supplementary material for: Transcriptomics of Haemophilus (Glässerella) parasuis serovar 5 subjected to culture conditions partially mimetic to natural infection for the search of new vaccine antigens
Source: BMC Vet Res. 2018 Nov 6;14:326. doi: 10.1186/s12917-018-1647-1 (PMC6219065; doi:10.1186/s12917-018-1647-1)
Supplement: Supplementary file 2 — Summary of genes that were downregulated under mimetic conditions with a log2 (fold change) > 10. Indicated findings in GenBank and Uniprot databases of genes that were downregulated under mimetic conditions with a log2 (fold change) > 10. P indicates that the encoded protein is related to pathogenesis, and NP indicates that there is no relationship with pathogenesis. The location of the protein is indicated with EX (extracellular), OM (outer membrane), PP (periplasmic), IM (inner membrane) or CP (cytoplasmic). * indicates that they are the same protein (PDF 127 kb). [file 12917_2018_1647_MOESM2_ESM.pdf]

Locus	Genbank product	no. Uniprot	Name Uniprot	Pathogeny	Localization
HAPS_RS00025	fumarate reductase flavoprotein subunit	B8F308	|frdA|Fumarate reductase flavoprotein subunit	NP	CP
HAPS_RS00030	succinate dehydrogenase/fumarate reductase iron-sulfur subunit	B8F309	|frdB|Succinate dehydrogenase iron-sulfur subunit	NP	PP
HAPS_RS00035	fumarate reductase subunit C	B8F310	|frdC|Fumarate reductase subunit C	NP	IM
HAPS_RS00040	fumarate reductase subunit D	B8F311	|frdD|Fumarate reductase subunit D	NP	IM
HAPS_RS00340	cytochrome bd oxidase subunit I	B8F369	|cydA|Cytochrome D ubiquinol oxidase, subunit I	NP	IM
HAPS_RS00345	cytochrome d ubiquinol oxidase subunit II	B8F370	|cydB|Cytochrome D ubiquinol oxidase, subunit II	NP	IM
HAPS_RS00505	fatty acid metabolism transcriptional regulator FadR	B8F398	|fadR|Fatty acid metabolism regulator protein	NP	CP
HAPS_RS00775	formate transporter FocA	B8F3E8	|focA|FNT family formate-nitrite transporter	NP	IM
HAPS_RS00795	transporter	B8F3F2	|HAPS_0158|Putative transport protein HAPS_0158	NP	IM
HAPS_RS00805	dethiobiotin synthase	B8F3F4	|bioD|ATP-dependent dethiobiotin synthetase BioD	NP	CP
HAPS_RS00810	membrane protein	B8F3F5	|HAPS_0161|Uncharacterized protein	NP	PP
HAPS_RS00945	tagatose-bisphosphate aldolase	B8F3I2	|lacD|Tagatose 1,6-diphosphate aldolase	NP	CP
HAPS_RS00950	PTS sugar transporter subunit IIA	B8F3I3	|ptsEIIA|PTS system, IIA component	NP	CP
HAPS_RS00955	beta-galactosidase	B8F3I4	|bgaC|Beta-galactosidase, glucosyl hydrolase family protein	NP	CP
HAPS_RS00960	PTS fructose transporter subunit IID	B8F3I5	|ptsEIID|PTS system, IIDcomponent	P	IM
HAPS_RS00965	PTS sugar transporter subunit IIC	B8F3I6	|ptsEIIC|PTS system, IIC component	P	IM
HAPS_RS00970	PTS mannose/fructose/sorbose transporter subunit IIB	B8F3I7	|ptsEIIB|PTS system, IIB component	NP	CP
HAPS_RS00975	tagatose-6-phosphate ketose isomerase	B8F3I8	|agaS|Tagatose-6-phosphate ketose/aldose isomerase	NP	CP
HAPS_RS00980	DeoR/GlpR transcriptional regulator	B8F3I9	|agaR|Transcriptional repressor AgaR/DNA-binding transcriptional dual regulator	NP	CP
HAPS_RS01175	maltose transporter permease	B8F3M4	|malG|ABC-type maltose transport systems, permease component	P	IM
HAPS_RS01180	maltose ABC transporter permease MalF	B8F3M5	|malF|ABC-type maltose transport systems, permease components	P	IM
HAPS_RS01190	ABC transporter ATP-binding protein	B8F3M7	|malK|Maltose/maltodextrin import ATP-binding protein MalK	NP	CP
HAPS_RS01320	tRNA-dihydrouridine synthase A			P	CP
HAPS_RS01325	pseudo				
HAPS_RS01410	sulfate ABC transporter substrate-binding protein	B8F3S0	|fdxG_2|Transcriptional formate dehydrogenase alpha major subunit/formate dehydrogenase, nitrate-inducible, major subunit	NP	PP

Locus	Genbank product	no. Uniprot	Name Uniprot	Pathogeny	Localization
HAPS_RS01415	formate dehydrogenase-N subunit alpha	B8F3S1	|fdxG_1|Transcriptional formate dehydrogenase alpha major subunit/formate dehydrogenase, nitrate-inducible, major subunit	NP	PP
HAPS_RS01420	formate dehydrogenase subunit beta	B8F3S2	|fdxH|Formate dehydrogenase iron-sulfur subunit, beta subunit/Fe-S-cluster-containing hydrogenase components 1	NP	CP
HAPS_RS01425	formate dehydrogenase subunit gamma	B8F3S3	|fdxI|Formate dehydrogenase cytochrome B556 subunit, gamma subunit	NP	IM
HAPS_RS01440	glycogen-branching enzyme	B8F3S5	|glgB|1,4-alpha-glucan branching enzyme GlgB	NP	CP
HAPS_RS01445	glycogen debranching enzyme	B8F3S6	|glgX|Glycogen operon protein GlgX	NP	CP
HAPS_RS01450	glucose-1-phosphate adenylyltransferase	B8F3S7	|glgC|Glucose-1-phosphate adenylyltransferase	NP	CP
HAPS_RS01780	cold shock domain protein CspD	B8F3Z0	|cspD|Cold shock-like protein CspD	NP	CP
HAPS_RS01970	hypothetical protein		|HAPS_0398|UPF0265 protein HAPS_0398	NP	CP
HAPS_RS02010	N-acetylneuraminate lyase	B8F434	|nanA|N-acetylneuraminate lyase	NP	CP
HAPS_RS02015	MurR/RpiR family transcriptional regulator	B8F435	|HAPS_0407|Putative HTH-type transcriptional regulator	NP	CP
HAPS_RS02020	YhcH/YjgK/YiaL family protein	B8F436	|HAPS_0408|Uncharacterized protein	NP	CP
HAPS_RS02180	galactose ABC transporter substrate-binding protein	B8F459	|mglB|Galactose ABC transporter periplasmic-binding protein/LacI transcriptional regulator	NP	PP
HAPS_RS02185	galactose/methyl galactoside ABC transporter ATP-binding protein MglA	B8F460	|mglA|Galactose/methyl galactoside import ATP-binding protein MglA	NP	CP
HAPS_RS02190	galactoside ABC transporter permease MglC	B8F461	|mglC|Galactose ABC transporter inner membrane component	NP	IM
HAPS_RS02290	carbon storage regulator	B8F480	|csrA|Carbon storage regulator homolog	NP	CP
HAPS_RS02300	universal stress protein A			NP	CP
HAPS_RS02505	MarC family protein	B8F4B7	|marC|UPF0056 inner membrane protein	P	IM
HAPS_RS02860	hypothetical protein	B8F4I2	|HAPS_0581|Hypothetical phage protein	NP	CP
HAPS_RS02875	hypothetical protein	B8F4I5	|HAPS_0584|Uncharacterized protein	NP	CP
HAPS_RS02885	hypothetical protein			NP	CP
HAPS_RS02890	phage tail protein/putative Fels-1 prophage host specificity protein	B8F4I8	|HAPS_0587|Phage tail protein/putative Fels-1 prophage host specificity protein	P	OM
HAPS_RS02905	tail assembly protein	B8F4J1	|HAPS_0590|Bacteriophage lambda tail assembly I	P	PP
HAPS_RS02960	hypothetical protein	B8F4K2	|HAPS_0601|Phage protein	P	EX
HAPS_RS02975	phage tail protein	B8F4K5	|HAPS_0604|Possible bacteriophage tail protein	NP	CP

Locus	Genbank product	no. Uniprot	Name Uniprot	Pathogeny	Localization
HAPS_RS02980	hypothetical protein	B8F4K6	|HAPS_0605|Putative RecA/RadA recombinase	NP	CP
HAPS_RS02985	peptidase S14	B8F4K7	|HAPS_0606|ATP-dependent Clp protease proteolytic subunit	NP	PP
HAPS_RS02990	phage portal protein	B8F4K8	|HAPS_0607|Bacteriophage capsid protein	NP	CP
HAPS_RS02995	hypothetical protein	B8F4K9	|HAPS_0608|Hypothetical prophage protein	NP	CP
HAPS_RS03175	ribosome-binding factor A	B8F4P5	|HAPS_0648|Putative serine protease	P	EX
HAPS_RS03195	2-oxoglutarate dehydrogenase subunit E1	B8F4P9	|kgd|Alpha-ketoglutarate decarboxylase	NP	CP
HAPS_RS03200	dihydrolipoamide succinyltransferase	B8F4Q1	|sucB|Dihydrolipoyllysine-residue succinyltransferase component of 2-oxoglutarate dehydrogenase complex	NP	CP
HAPS_RS03205	succinyl-CoA ligase subunit beta	B8F4Q2	|sucC|Succinate--CoA ligase [ADP-forming] subunit beta	NP	CP
HAPS_RS03210	succinate--CoA ligase subunit alpha	B8F4Q3	|sucD|Succinate--CoA ligase [ADP-forming] subunit alpha	NP	CP
HAPS_RS03310	lactate utilization protein C	B8F4S4	|HAPS_0678|Putative conserved iron-sulfur protein	NP	CP
HAPS_RS03315	iron-sulfur cluster-binding protein	B8F4S5	|HAPS_0679|Iron-sulfur cluster binding reductase	NP	CP
HAPS_RS03320	hypothetical protein	B8F4S6	|HAPS_0680|Fe-S oxidoreductase	NP	CP
HAPS_RS03325	lactate permease	B8F4S7	|lctP|L-lactate permease	NP	IM
HAPS_RS03495	Derived by automated computational analysis using gene prediction method: Protein Homology	B8F4W1	|pckA|Phosphoenolpyruvate carboxykinase (ATP)	NP	CP
HAPS_RS03660	DNA starvation/stationary phase protection protein	B8F4Z3	|dps|Ferritin and DNA-binding stress protein	P	CP
HAPS_RS03685	superoxide dismutase	B8F4Z8	|sodC|Superoxide dismutase [Cu-Zn]	P	PP
HAPS_RS04120	transporter	B8F573	|comEA|DNA uptake protein	P	CP
HAPS_RS04145	phosphomannomutase	B8F578	|manB|Phosphomannomutase	NP	PP
HAPS_RS04150	rRNA methyltransferase	B8F579	|ftsJ|rRNA methyltransferase	NP	CP
HAPS_RS04170	beta-ketoacyl-ACP reductase	B8F583	|fabG|3-ketoacyl-(Acyl-carrier-protein) reductase	NP	CP
HAPS_RS04655	PTS glucose transporter subunit IIA	B8F5I2	|crr|Glucose-specific PTS system component	NP	CP
HAPS_RS04660	phosphoenolpyruvate--protein phosphotransferase	B8F5I3	|ptsI|Phosphoenolpyruvate-protein phosphotransferase	NP	CP
HAPS_RS04665	HPr family phosphocarrier protein	B8F5I4	|ptsH|Phosphotransferase system, phosphocarrier protein HPr	NP	CP
HAPS_RS04705	preprotein translocase subunit YajC	B8F5J1	|yajC|Preprotein translocase subunit YajC	NP	CP
HAPS_RS04720	2,3-bisphosphoglycerate-dependent phosphoglycerate mutase	B8F5J4	|gpmA|2,3-bisphosphoglycerate-dependent phosphoglycerate mutase	NP	CP
HAPS_RS04740	transketolase	B8F5J8	|tktA|Transketolase	NP	CP
HAPS_RS04770	malate dehydrogenase	B8F5K4	|mdh|Malate dehydrogenase	NP	CP

Locus	Genbank product	no. Uniprot	Name Uniprot	Pathogeny	Localization
HAPS_RS04820	hypothetical protein	B8F5K9	|HAPS_0991|Uncharacterized protein	NP	CP
HAPS_RS04825	5S ribosomal RNA				
HAPS_RS04845	ABC transporter substrate-binding protein	B8F5L3	|oppA|Oligopeptide permease ABC transporter membrane protein	P	PP
HAPS_RS04905	PTS sucrose transporter subunit IIBC	B8F5M5	|ptsB|PTS system sucrose-specific EIIBC component	NP	IM
HAPS_RS05085	peptidylprolyl isomerase	B8F5R4	|slyD|Peptidyl-prolyl cis-trans isomerase	NP	CP
HAPS_RS05160	arginine transporter permease subunit ArtM	B8F5S9	|artM|Arginine transporter permease subunit ArtM	P	IM
HAPS_RS05165	arginine transporter permease subunit ArtQ			P	IM
HAPS_RS05170	arginine ABC transporter substrate-binding protein	B8F5T1	|artI|ABC-type amino acid transport/signal transduction systems, periplasmic component/domain	NP	PP
HAPS_RS05175	arginine ABC transporter ATP-binding protein ArtP	B8F5T2	|artP|ABC-type arginine transport system, ATPase component	NP	CP
HAPS_RS05280	ammonia-forming cytochrome c nitrite reductase subunit c552	B8F5V4	|nrfA|Cytochrome c-552	NP	PP
HAPS_RS05285	cytochrome c nitrite reductase pentaheme subunit	B8F5V5	|nrfB|Cytochrome c-type protein NrfB	NP	PP
HAPS_RS05290	cytochrome c nitrite reductase Fe-S protein	B8F5V6	|nrfC|Nitrate reductase	NP	PP
HAPS_RS05295	cytochrome c nitrite reductase subunit NrfD	B8F5V7	|nrfD|Nitrate reductase, transmembrane protein	NP	IM
HAPS_RS05325	metal ABC transporter substrate-binding protein	B8F5W2	|yfeA|Chelated iron ABC transporter, periplasmic-binding protein	P	PP
HAPS_RS05385	aspartate ammonia-lyase	B8F5X1	|aspA|Aspartate ammonia-lyase	NP	CP
HAPS_RS05500	cytochrome-c peroxidase	B8F5Z1	|ccp|Cytochrome c peroxidase	NP	PP
HAPS_RS05665	cell division protein ZapA	B8F622	|zapA|Cell division protein ZapA	NP	CP
HAPS_RS05790	tellurite resistance methyltransferase TehB	B8F643	|tehB|Tellurite resistance protein TehB	NP	CP
HAPS_RS05835	fructose-bisphosphatase class I	B8F651	|fbp|Fructose-1,6-bisphosphatase class 1	NP	CP
HAPS_RS05895	GTPase HflX	B8F664	|hflX|GTPase HflX	NP	CP
HAPS_RS05905	N-acetylmannosamine kinase	B8F666	|nanK|N-acetylmannosamine kinase	NP	CP
HAPS_RS05910	N-acetylmannosamine-6-phosphate 2-epimerase	B8F667	|nanE|Putative N-acetylmannosamine-6-phosphate 2-epimerase	NP	CP
HAPS_RS05970	tRNA-Gly				
HAPS_RS05975	tRNA-Tyr				
HAPS_RS05980	tRNA-Thr				
HAPS_RS06050	tRNA-Leu				
HAPS_RS06460	tRNA-His				
HAPS_RS06465	tRNA-Pro				
HAPS_RS06500	bifunctional proline dehydrogenase/L-glutamate gamma-semialdehyde dehydrogenase	B8F6G8	|putA|Bifunctional protein PutA	NP	CP
HAPS_RS06680	serine protease	B8F6K6	|espP1|Putative extracellular serine protease (Autotransporter)	P	EX

Locus	**Genbank product**	**no. Uniprot**	**Name Uniprot**	**Pathogeny**	**Localization**
HAPS_RS06850	phosphopantetheine adenylyltransferase	B8F6N2	|coaD|Phosphopantetheine adenylyltransferase	NP	CP
HAPS_RS07095	tRNA-Leu				
HAPS_RS07160	serine peptidase	B8F6T4	|htrA|Periplasmic serine protease do/hhoA-like	NP	PP
HAPS_RS07290	anaerobic C4-dicarboxylate transporter			P	IM
HAPS_RS07415	L-fucose isomerase	B8F6Y0	|fucI|L-fucose isomerase	NP	CP
HAPS_RS07420	L-fuculokinase	B8F6Y1	|fucK|L-fuculokinase	NP	OM
HAPS_RS07425	L-fucose mutarotase	B8F6Y2	|fucU|L-fucose mutarotase	NP	CP
HAPS_RS07440	5-dehydro-2-deoxygluconokinase	B8F6Y5	|iolC|Myoinositol catabolism protein, sugar kinase	NP	CP
HAPS_RS07445	3D-(3,5/4)-trihydroxycyclohexane-1,2-dione acylhydrolase (decyclizing)	B8F6Y6	|iolD|Myoinositol catabolism protein, acetolactate synthase	NP	CP
HAPS_RS07450	myo-inosose-2 dehydratase	B8F6Y7	|iolE|Inosose dehydratase	NP	CP
HAPS_RS07460	PTS fructose transporter subunit IIC	B8F6Y9	|celB|Permease IIC component	NP	IM
HAPS_RS07465	alpha-L-fucosidase	B8F6Z0	|alfA|Alpha-L-fucosidase	NP	CP
HAPS_RS07470	gfo/Idh/MocA family oxidoreductase	B8F6Z1	|HAPS_1537|Dehydrogen ase	NP	CP
HAPS_RS07475	ABC transporter permease			P	IM
HAPS_RS07480	sugar ABC transporter ATP-binding protein	B8F6Z3	|yphE|Fused predicted sugar transporter subunits of ABC superfamily: ATP-binding components	NP	CP
HAPS_RS07485	ATPase	B8F6Z4	|yphF|Predicted sugar transporter subunit: periplasmic-binding component of ABC superfamily	NP	CP
HAPS_RS07490	methylmalonate-semialdehyde dehydrogenase (CoA acylating)	B8F6Z5	|HAPS_1541|Methylmalo nate semialdehyde dehydrogenase	NP	CP
HAPS_RS07640	DNA processing protein DprA	B8F725	|smf|Smf protein, Rossmann fold nucleotide-binding protein involved in DNA uptake	NP	CP
HAPS_RS07765	class II fumarate hydratase	B8F751	|fumC|Fumarate hydratase class II	NP	PP
HAPS_RS07830	sialidase			P	OM
HAPS_RS07850	F0F1 ATP synthase subunit I	B8F767	|atpI|F0F1 ATP synthase subunit I/H(+)-transporting two-sector ATPase, F(0) subunit I	NP	IM
HAPS_RS07890	F0F1 ATP synthase subunit epsilon	B8F775	|atpC|ATP synthase epsilon chain	NP	CP
HAPS_RS07900	D-ribose pyranase	B8F777	|rbsD|D-ribose pyranase	NP	CP
HAPS_RS07905	ribose ABC transporter ATP-binding protein RbsA	B8F778	|rbsA1|Ribose import ATP-binding protein RbsA	NP	CP
HAPS_RS07910	ribose ABC transporter permease	B8F779	|rbsC1|ABC D-ribose transporter permease protein RbsC	NP	IM
HAPS_RS07915	D-ribose ABC transporter substrate-binding protein	B8F780	|rbsB1|ABC D-ribose transporter periplasmic-binding compoent RbsB	NP	PP
HAPS_RS07920	ribokinase	B8F781	|rbsK1|Ribokinase	NP	CP

Locus	Genbank product	no. Uniprot	Name Uniprot	Pathogeny	Localization
HAPS_RS08025	paraslipin	B8F796	|hflC|SPFH domain-containing protein	NP	IM
HAPS_RS08030	NfeD family protein	B8F797	|HAPS_1654|Regulator of membrane protease activity	NP	IM
HAPS_RS08355	hypothetical protein	B8F7E0	|HAPS_1720|Uncharacterized protein	NP	PP
HAPS_RS08380	LacI family transcriptional regulator	B8F7E3	|rbsB2|ABC D-ribose transporter periplasmic-binding compoent RbsB	NP	PP
HAPS_RS08385	ABC transporter permease	B8F7E4	|rbsC2|ABC D-ribose transporter permease protein RbsC	P	IM
HAPS_RS08405	PTS mannose transporter subunit IID	B8F7E8	|manZ|PTS system mannose-specific protein IID	P	IM
HAPS_RS08410	PTS mannose/fructose/sorbose transporter subunit IIC	B8F7E9	|manY|PTS system mannose/fructose/sorbose family IIC subunit	P	IM
HAPS_RS08415	PTS mannose transporter subunit EIIAB	B8F7F0	|manX|PTS system mannose-specific EIIAB component	NP	CP
HAPS_RS08450	ATP-dependent protease	B8F7F8	|comM|Competence protein M	P	CP
HAPS_RS08485	triose-phosphate isomerase	B8F7G2	|tpiA|Triosephosphate isomerase	NP	CP
HAPS_RS08625	hypothetical protein	B8F7I4	|HAPS_1774|Uncharacterized protein	P	PP
HAPS_RS08630	hypothetical protein			NP	CP
HAPS_RS08660	PTS ascorbate transporter subunit IIA	B8F7I8	|ulaC|Ascorbate-specific phosphotransferase protein IIA component, Phosphotransferase system mannitol/fructose-specific IIA domain (Ntr-type)	NP	CP
HAPS_RS08665	PTS ascorbate transporter subunit IIBC	B8F7I9	|ulaA|Ascorbate-specific PTS system enzyme IIC/IIB	NP	IM
HAPS_RS08705	hypothetical protein	B8F7J4	|mazG|Nucleotide pyrophosphohydrolase	NP	CP
HAPS_RS08725	cytochrome c	B8F7J8	|napC|Cytochrome c-type protein	NP	PP
HAPS_RS08730	nitrate reductase cytochrome C550 subunit	B8F7J9	|napB|Periplasmic nitrate reductase, electron transfer subunit	NP	PP
HAPS_RS08735	quinol dehydrogenase ferredoxin subunit NapH	B8F7K0	|napH|Quinol dehydrogenase membrane component, NapH/MauN family ferredoxin-type protein	NP	IM
HAPS_RS08740	ferredoxin-type protein NapG			NP	PP
HAPS_RS08745	periplasmic nitrate reductase subunit alpha	B8F7K2	|napA|Periplasmic nitrate reductase	NP	PP
HAPS_RS08750	reductase	B8F7K3	|napD|Periplasmic nitrate reductase subunit NapD	P	CP
HAPS_RS08755	ferredoxin-type protein NapF			NP	CP
HAPS_RS08760	50S ribosomal protein L36	B8F7K6	|rpmJ1|50S ribosomal protein L36	NP	CP
HAPS_RS08765	50S ribosomal protein L31 type B	B8F7K7	|rpmE2|50S ribosomal protein L31 type B	NP	CP
HAPS_RS08770	ribulose-phosphate 3-epimerase	B8F7K8	|rpe|Ribulose-phosphate 3-epimerase	NP	CP
HAPS_RS08775	arylsulfatase	B8F7K9	|HAPS_1801|Arylsulfatase A	NP	CP

Locus	Genbank product	no. Uniprot	Name Uniprot	Pathogeny	Localization
HAPS_RS08780	solute:sodium symporter family transporter	B8F7L0	|yidK|SSS family solute:sodium (Na+) symporter	NP	IM
HAPS_RS08825	lactate dehydrogenase	B8F7L9	|HAPS_1811|Malate/L-lactate dehydrogenase	NP	CP
HAPS_RS08830	hypothetical protein			NP	CP
HAPS_RS08970	metalloprotease TldD	B8F7P5	|tldD|Protease involved in Microcin B17 maturation and in sensitivity to the DNA gyrase inhibitor LetD	NP	CP
HAPS_RS09005	pseudo				
HAPS_RS09185	pseudo				
HAPS_RS09225	C4-dicarboxylate ABC transporter substrate-binding protein	B8F7T5	|HAPS_1899|TRAP transporter solute receptor TAXI family protein	P	PP
HAPS_RS09230	C4-dicarboxylate ABC transporter	B8F7T6	|dctM|TRAP C4-dicarboxylate transport system permease DctM subunit	P	IM
HAPS_RS09235	universal stress protein	B8F7T7	|uspA|Universal stress protein A	NP	CP
HAPS_RS09295	alanine dehydrogenase	B8F7U9	|ald|Alanine dehydrogenase	NP	CP
HAPS_RS09300	sodium-dependent transporter	B8F7V0	|HAPS_1914|Transporter	NP	IM
HAPS_RS09530	uridine phosphorylase	B8F7Y9	|udp|Uridine phosphorylase	NP	CP
HAPS_RS09535	DUF413 family protein	B8F7Z0	|HAPS_1962|Uncharacterized protein	NP	CP
HAPS_RS09610	acid phosphatase/phosphotransferase	B8F802	|napA|Class B acid phosphatase	NP	PP
HAPS_RS09770	prepilin-type cleavage/methylation domain-containing protein	B8F829	|pilA|Tfp pilus assembly protein, major pilin PilA	P	EX
HAPS_RS09940	DNA repair protein RadC			NP	CP
HAPS_RS10105	pseudo				
HAPS_RS10345	transporter	B8F8C4	|HAPS_2130|Di-and tricarboxylate transporter	NP	IM
HAPS_RS10570	bifunctional malic enzyme oxidoreductase/phosphotransacetylase	B8F8F9	|sfcA|Malic enzyme	NP	CP
HAPS_RS10920	beta-D-galactosidase			NP	EX
HAPS_RS11050	isocitrate dehydrogenase (NADP(+))	B8F8Q3	|icd|Isocitrate dehydrogenase [NADP]	NP	CP
HAPS_RS11055	Derived by automated computational analysis using gene prediction method: Protein Homology	B8F8Q4	|acnB|Aconitate hydratase B	NP	CP
HAPS_RS11060	citrate (Si)-synthase	B8F8Q5	|gltA|Citrate synthase	NP	CP
HAPS_RS11095	hypothetical protein	B8F8R2	|comA|Competence protein A	NP	CP
HAPS_RS11100	hypothetical protein	B8F8R3	|HAPS_2286|Uncharacterized protein	P	PP
HAPS_RS11105	hypothetical protein	B8F8R4	|HAPS_2287|Chromosome segregation ATPase	P	EX
HAPS_RS11110	hypothetical protein	B8F8R5	|HAPS_2288|Uncharacterized protein	P	PP
HAPS_RS11115	secretin	B8F8R6	|comE|Competence protein E/type II secretory pathway, component HofQ	P	OM
